# Stepping down inhaled corticosteroid use in asthma - who is responsible?

**DOI:** 10.1186/2045-7022-5-S2-P2

**Published:** 2015-03-23

**Authors:** Ashbal Chaudhary, Umair Gondal, Valeed Ghafoor

**Affiliations:** 1 University of Manchester, Medical School, Manchester, UK; 2 Royal Preston Hospital, Renal Medicine, Preston, UK

## 

Asthma is a very common condition affecting 5.4 million people in the United Kingdom. Daily use of inhaled corticosteroids (ICS) prevents acute exacerbations and reduces need of other inhalers (ie Salbutamol). They are however amongst the most expensive prescription medicines in the UK, bearing a substantial financial burden on the NHS. ICS use is also linked with a higher risk of side effects such as developing oral thrush.

Guidelines published by NICE stress that apt patient education is one necessary component of managing asthma, to increase adherence to treatment and subsequently improve symptomatic control. To assess the degree of adherence to ICS treatment, the files of all asthmatic patients currently prescribed ICS were analyzed, focusing on regularity of dispensing medicines.

The results showed that the vast majority of patients were using their ICS inhaler in an ad-hoc fashion and not as prescribed; concluding that these patients did not require their ICS inhalers (either entirely, or at their current dosages). A significant cause of this was identified as being a lack of, or poor use of, communication between the practitioner and patient at the time of prescribing.

In conclusion, insufficient patient education at time of starting medicines leads to poor adherence to treatment and causes financial inefficiencies regarding prescribing, for any given practice. Patient understanding must always be gauged, at the time of starting ICS treatment, and thereon, during subsequent consultations. To aid this process, a patient information leaflet should be used as means of signposting for the practitioner and as a treatment record for the patient.

